# Towards an Electronic Dog Nose: Surface Plasmon Resonance Immunosensor for Security and Safety

**DOI:** 10.3390/s140916586

**Published:** 2014-09-05

**Authors:** Takeshi Onodera, Kiyoshi Toko

**Affiliations:** 1 Research and Development Center for Taste and Odor Sensing, Kyushu University, 744 Motooka, Nishi-ku, Fukuoka 819-0395, Japan; E-Mail: toko@ed.kyushu-u.ac.jp; 2 Graduate School of Information Science and Electrical Engineering, Kyushu University, 744 Motooka, Nishi-ku, Fukuoka 819-0395, Japan

**Keywords:** electronic dog nose, surface plasmon resonance, immunosensor, antibody, explosives, security and safety

## Abstract

This review describes an “electronic dog nose” based on a surface plasmon resonance (SPR) sensor and an antigen–antibody interaction for security and safety. We have concentrated on developing appropriate sensor surfaces for the SPR sensor for practical use. The review covers different surface fabrications, which all include variations of a self-assembled monolayer containing oligo(ethylene glycol), dendrimer, and hydrophilic polymer. We have carried out detection of explosives using the sensor surfaces. For the SPR sensor to detect explosives, the vapor or particles of the target substances have to be dissolved in a liquid. Therefore, we also review the development of sampling processes for explosives, and a protocol for the measurement of explosives on the SPR sensor in the field. Additionally, sensing elements, which have the potential to be applied for the electronic dog nose, are described.

## 1. Introduction

Working dogs have been deployed for use in various places, such as the police force, airport customs, disaster-affected areas, snowy mountains, and the home. Dogs can find a given target using their excellent sense of smell. A dog's sensitivity to odor has been expressed well in a quote from Horowitz [1], who states that “As we see the world, the dog smells it”.

Since the terrorist attacks in the USA on 11 September 2001, concern about terrorist attacks has also increased in Japan. Dogs trained to detect explosives were deployed at Narita airport in Japan in preparation for holding the 2002 FIFA World Cup. A dog's sensitivity depends on its physical condition, and a dog cannot be employed to search for explosives for a prolonged time [2]. The training of dogs is costly, and dog handlers also need extensive training [3,4]. When the Great East Japan Earthquake occurred, search and rescue dogs were sent to the devastated area to find trapped humans. However, the number of search and rescue dogs is insufficient for large-scale disasters. Therefore, electronic dog noses, which play the role of a dog's nose, are desired. We describe an electronic dog nose based on a surface plasmon resonance (SPR) sensor, an antigen–antibody interaction and a sampling method for hazardous substances. Additionally, sensing elements, which have the potential to be applied for the electronic dog nose, are reviewed.

## 2. Electronic Dog Nose Based on Surface Plasmon Resonance Biosensor

In general, a dog's olfactory sense related to explosive detection functions briefly as follows. The odors come into contact with the sensory organs most efficiently through the act of sniffing. The odor compounds are dissolved in the olfactory mucus layers within the nasal cavity, where the epithelium contains the bulk of the olfactory receptors. Interaction between the odors and the appropriate receptors causes a second messenger cascade. The second messenger then elicits a receptor potential by opening sodium channels, eventually to the point of causing an action potential. The action potentials reach the brain through the neurons of the olfactory nerve to a variety of sub-cortical and cortical structures for further encoding and, ultimately, perception [2]. As reported [5], the limit of detection of a dog to 2,4-dinitrotoluene (DNT) was in the 500 ppt range.

**Figure 1.**
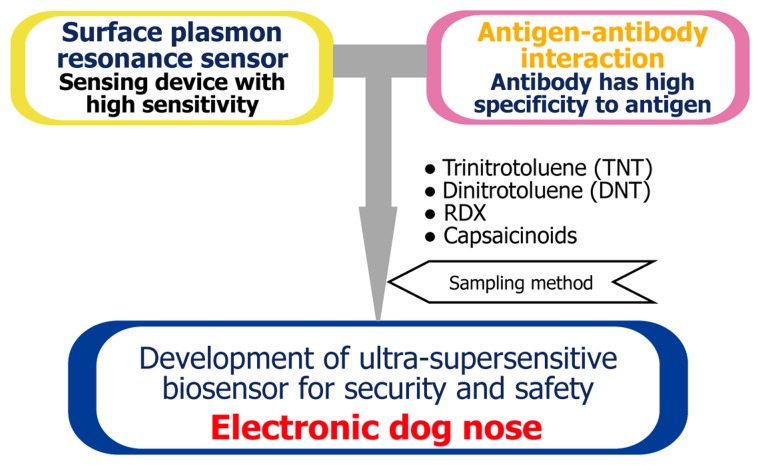
Composition of an electronic dog nose.

Figure 1 shows the composition of the electronic dog nose, in which an SPR sensor is utilized as the transducer. The sensitivity for changes of the dielectric constant near the surface is high and the system size can be small. An antigen–antibody interaction is adopted for the highly selective sensing of chemical substances. In this case, an antibody is used instead of an olfactory receptor. Chemical compounds need to be in a buffer solution for antigen–antibody interactions to occur. Explosives thus have to be dissolved for measurement using the SPR sensor and to induce the antigen–antibody interaction regardless of the sampling method.

The principle of the SPR sensor is briefly as follows. An evanescent wave appears on the surface of a thin metal film, such as a Au or a Ag film, on a prism, when light is focused onto the film from the prism side at an incidence angle that results in total reflection. Surface plasmons on the surface of the metal film facing the sample solution are excited by the evanescent wave. When the wave numbers of the evanescent wave and the surface plasmon are equal, the intensity of refraction is reduced because the energy of photon is transferred to the SPR. The resonance angle, which is the incident angle at which the refraction intensity is at its minimum, is determined by the refraction indices of the thin film and attached solution. When the binding of an antigen to an antibody immobilized on a thin film occurs, the resonance angle is markedly changed. The SPR sensor is a type of refractive index detector [6–9].

Selectivity of antibodies is very important in this concept. Zeck *et al.* have reported that a commercially available monoclonal antibody against 2,4,6-trinitrotoluene (TNT) (Strategic biosolutions, clone TNT A/1.1.1; TNT–Ab) rarely reacts with compounds other than TNT, which shares similar chemical structures [10]. TNT-like compounds have aromatic rings and nitro functional groups and the antibody shows very low cross-reactivities to them. We have tried to develop antibodies against explosives [11–16]. However, we have consistently used the commercial antibody to compare newly modified sensor surfaces for detection of TNT.

An SPR immunosensor has the capability of detecting low molecular weight compounds such as explosives, chemical warfare agents (CWAs), and biological macromolecules, such as toxic proteins and viruses on the same system [17–19]. Thus, SPR immunosensors enables on-site detection of these hazardous substances.

Some excellent review papers have been published for explosive detection methods [20–22]. Review papers on natural olfactry receptors-integrated sensors [23] and sensing of small molecule by an SPR immunosensor [24] have also been published. Therefore, we described this review paper from the perspective of how to establish the function of dog noses artificially for the detection of hazardous compounds, mainly explosives. The limit of detections (LODs) for hazardous compounds and the conditions of SPR sensing are summarized in Section 4.

## 3. Assay Type: Indirect Competitive Assay

**Figure 2.**
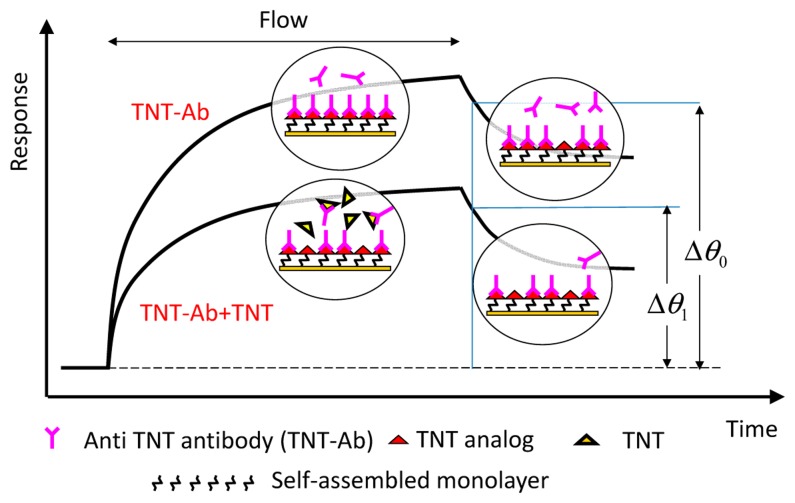
Principle of indirect competitive assay.

Indirect competitive assay (inhibition assay) is used for expanding the shift in the resonance angle in measurements of molecular interaction on the SPR sensor. Figure 2 shows the principle of the indirect competitive method [3]. In the method, antibodies that are bound to immobilized antigen analogs on an Au surface are detected instead of antigens bound (target substances) to immobilized antibody on an Au surface [17,25,26]. Therefore, target substances can be detected indirectly and sensitively because of the difference between the molecular weights of the target substance and antibody, which is at least two orders of magnitude. Binding percentage, *i.e.*, the ratio of Δθ_1_ to Δθ_0_, was used as a parameter for evaluation [16]. An incubation time (binding time in a tube without pump flow) of 15–20 min to allow the reaction between the antibody and antigen (target substance) to occur is normally carried out before measurement.

TNT and DNT were measured with indirect competitive assay using SPR immunosensors with surfaces containing an ethylene glycol chain. An oligo(ethylene glycol) (OEG) chain is applied to surfaces to avoid non-specific adsorption of protein owing to its flexibility and hydrophilic property [27–29]. It is important to suppress non-specific adsorption to ensure highly sensitive detection. Authors reported a multistep method to form a mixed self-assembled monolayer (SAM) for easily controlling the ratio of terminal groups and enhancing the ability to suppress non-specific adsorption to the sensor surface [30]. For example, the carboxyl or amino group as a terminal group is used for immobilizing ligand or target analog, and the hydroxyl group is used for reducing non-specific adsorption. In the first step, SAM was formed on an Au thin film uniformly. Then, in the second step, a mixture of two types of linkers with arbitrary ratio was reacted with the terminal groups of the SAM. Finally, target analog was immobilized with the reactive group such as the amine group of the linker immobilized on the SAM. Immunoreaction occurs on these hydrophilic sensor surfaces and the surfaces with an antibody play the role of an olfactory receptor.

### 3.1. Target: 2,4-Dinitrotoluene (DNT)

#### Surface Functionalization: Oligo(ethylene glycol) (OEG)

**Scheme 1.**
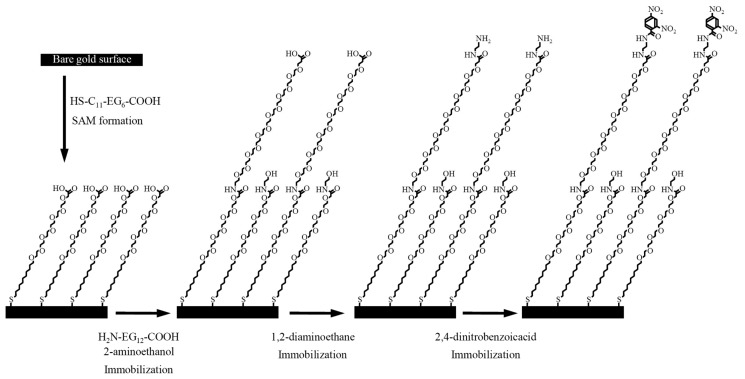
The fabrication process of the sensor chip for DNT detection [13].

Scheme 1 shows the fabrication process of the surface with a SAM containing OEG chains [13]. Firstly, the Au sensor surface was immersed in 2 mM HS-C_11_-EG_6_-COOH for 24 h after cleaning of a sensor chip. The sensor chip was immersed in a 1:1 mixture of 0.4 M *N*-ethyl-N′-(3-dimethylaminopropyl) carbodiimide (EDC; in water) and 0.1 M *N*-hydroxysuccinimide (NHS; in water) to activate the carboxyl group for 2 h. The sensor chip was then immersed in a 1:9 mixture of 10 mM H_2_N-EG_12_-COOH [in 25 mM borate buffer (pH 8.5)] and 10 mM 2-aminoethanol in 25 mM borate buffer (pH 8.5). The sensor chip was immersed in a 1 M aminoethanol-HCl solution (pH 8.5) for 10 min to block any NHS activated site. Then, the sensor chip was mounted onto a SPR sensor system (BiacoreJ). EDC/NHS solution was allowed to flow over both flow channels for activating of carboxyl group. Then, 0.1 M diaminoethane in borate buffer (pH 8.5) was injected for 20 min to introduce the amino group into the terminal of H_2_N-EG_12_-COOH. The other channel was deactivated with aminoethanol for use as a reference channel. A NHS-activated 2,4-dinitrobenzoic acid (DNBA) solution was dropped on the surface of the sensor chip. From the cross-reactivity data of a polyclonal antibody raised in a rabbit against dinitrophenylated keyhole limpet hemocyanin (DNP–KLH) for nitroaromatic compounds, DNBA was chosen as a suitable DNT analog [13]. An indirect competitive assay was subsequently conducted using the sensor chip and the polyclonal antibody. The assay exhibited a LOD of 20 pg/mL (20 ppt).

### 3.2. Target: 2,4,6-Trinitrotoluene (TNT)

#### 3.2.1. Surface Functionalization: Dendrimer

TNT concentration was measured using an SPR immunosensor with a dendrimer-modified SAM surface. The dendrimer repeatedly branches from the core molecule, and the number of repeated branching cycles is called the generation [31,32]. Polyamidoamine (PAMAM) dendrimers have been known to be used as building blocks for molecularly organized nanostructures. Monolayers fabricated with dendrimer on metallic surfaces and dendrimer-modified gold nanoparticle were reported for molecular interaction studies [33,34]. Multistep immobilization of proteins and DNA using dendrimers on gold films modified with alkanethiol SAM was reported [35]. Immobilization capacity of proteins on the chips was increased, compared to alkanethiol SAM surface without dendrimer layer. Another advantage of using this molecule is that chemical reactivity of functional group on the surface of dendrimer is higher than when present in other molecules [36]. A higher generation of a dendrimer molecule has more functional groups on its surface. We can produce binding sites for antibodies with a high density on a sensor surface using dendrimer.

**Figure 3.**
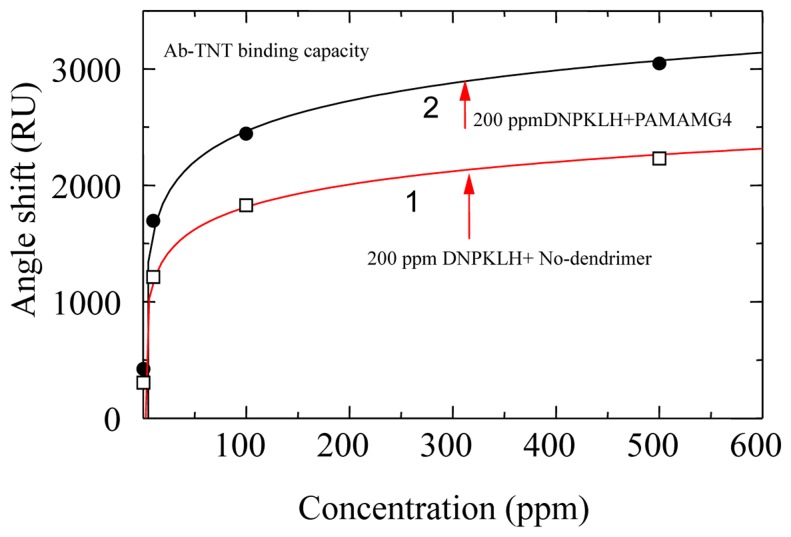
Dependence of resonance angle shift on dendrimer modified and control surfaces with antibody concentration [37].

We used a PAMAM–amidoethylethanol dendrimer, 1,12-diaminododecane core. Dendron arms radiated from the core. The PAMAM dendrimer was used to enhance of binding capacity of the antibody in our proposed sensor [37]. The Au sensor surface was first modified with an SAM using 11-amino-1-undecanethiol. The sensor surface was activated by a bis-sulfosuccinimidyl substrate (BS_3_); BS_3_ is a homobifunctional crosslinker that reacts with amine groups at both ends. The sensor surface was immersed in a 10% methanol solution of the PAMAM dendrimer generation 4 (G4) for 16–20 h. The immobilized dendrimer was treated with BS_3_ followed by 200 ppm DNP–KLH in phosphate buffered saline (PBS). Figure 3 shows a comparison of binding capacity for TNT–Ab on the sensor chip and a sensor chip without dendrimer. Binding capacity of the sensor chip modified with dendrimer is 1.6 times higher than that without dendrimer. The binding efficiency of TNT–Ab to the dendrimer surface may be due to the following reasons: a more accessible area was provided by the dendrimer molecules on the sensor surface; and the large number of available amine groups the subsequent coupling reaction was created by a steric arrangement of dendrimer molecules.

Indirect competitive assay for TNT was conducted on three different sensor chips. 500 ppb TNT–Ab was made to flow over the sensor surface. The amount of binding was approximately 480 RU. Approximately 370 RU was obtained by flow of 500 ppb TNT–Ab + 500 ppt TNT. The sensor response after the second injection of 500 ppb TNT–Ab was almost the same as that after the first injection of TNT–Ab solution. The obtained LOD for TNT on the sensor surface was 110 ± 50 (standard deviation: SD) ppt for three different chips.

#### 3.2.2. Surface Functionalization: OEG

**Figure 4.**
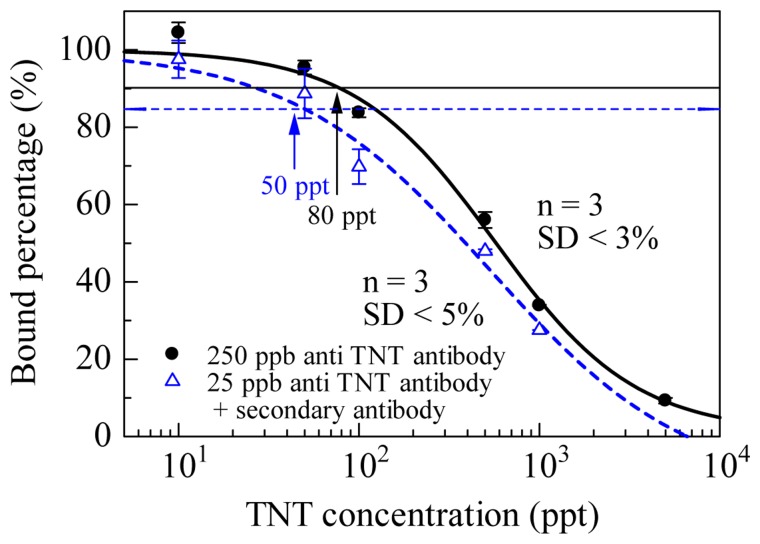
Response curves for TNT [4].

The solid line in Figure 4 shows the response curve for TNT using a sensor chip modified with mixed SAM, which was made with multisteps using an 8:2 mixture of 10 mM mono-*N*-t-boc-amido-dPEG_11_-amine and amino-dPEG_4_-alcohol in 10 mM disodium tetraborate buffer (1 M NaCl, pH 8.5) on a SAM formed with PEG_6_-COOH aromatic dialkanethiol [4,30]. 2,4-dinitrophenyl acetic acid (DNP–acetic acid), 2,4-dinitrophenyl glycine (DNP–gly), 2,4,6-trinitrophenyl glycine (TNP–gly) were immobilized on the three sensor chips modified with mono-*N*-t-boc-amido-dPEG_11_-amine after removing tert-butoxycarbonyl (t-boc) group for deprotection of amino group. As a result of affinity analysis, the affinity between DNP–acetic acid and anti-TNT–Ab was lower than that between TNP–gly or DNP–gly and anti-TNT–Ab. Zeck *et al.* reported that cross-reactivity of the antibody to dinitroaromatic compounds were below 6% [10]. However, TNT–Ab binding to the DNP–acetic acid chip was observed. The vertical axis indicates that the ratio of the binding amount of TNT–Ab without TNT and TNT–Ab with TNT. The LOD of 80 ppt was achieved and IC_50_ (half maximal inhibitory concentration) was 574 ppt. SD were <3% at each TNT concentration. SDs were very small, and reproducible results were obtained. The LOD was based on 3SD (*ca.* 10%) of blank (binding of TNT–Ab without TNT) and decided upon as the concentration of TNT at 90% antibody binding. The sensor chip also exhibited the ability to avoid non-specific adsorption of high concentration of protein solution.

Improvement of the LOD to TNT was attempted by using secondary antibody. Anti-mouse IgG antibody (from rabbit; secondary antibody) was allowed to flow over the sensor surface after injection of TNT–Ab (primary antibody) or TNT–Ab with TNT. Secondary antibody would interact with TNT–Ab bound on the sensor surface. Sensor response would be obtained to calculate bound percentage sufficiently, because the sensor response composes the binding of the primary antibody bound on the sensor surface and the binding of the secondary antibody to the primary antibody. Consequently, the concentration of TNT–Ab can be reduced. Dashed line in the Figure 4 shows the response curve. The concentration of TNT–Ab and secondary antibody were 25 ppb and 100 ppm, respectively. LOD of 50 ppt for TNT was obtained. This result enabled the use of SAM containing the OEG, which suppressed non-specific adsorption. The sensor surface can be used repeatedly for more than 100 cycles of measurement. LOD to TNT was improved using secondary antibody; however, SD was also enlarged on each measurement. Therefore, significant improvement of the sensitivity enhancement was not obtained.

#### 3.2.3. Surface Functionalization: Dendron

**Scheme 2.**
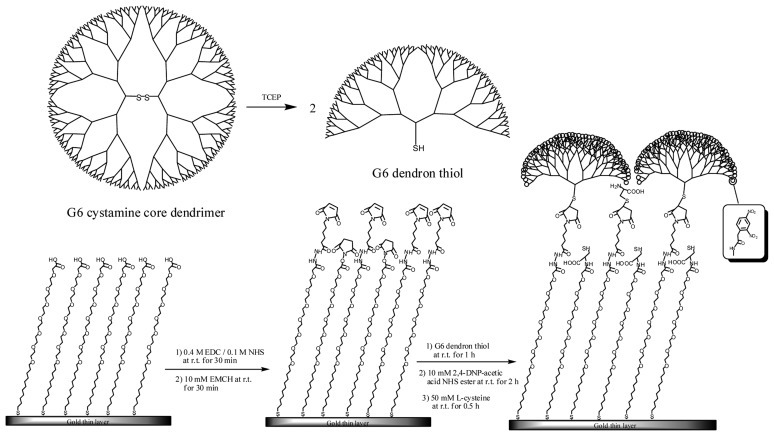
The fabrication procedures of the G6-dendron-based sensor surface [39].

Effectiveness of the dendrimer and the SAM surface containing OEG chains for SPR measurement was described above (Sections 3.2.1 and 3.2.2). The accessibility to the target biological molecule can be enhanced by the steric structure of dendrimers and their high density of binding sites [35,38]. A new sensor surface was fabricated combining these merits for achieving lower LOD. The fabrication procedure of the dendron-immobilized sensor surface is shown in Scheme 2 [39]. Cystamine-core PAMAM dendrimers can be cleaved into two molecules, thiol-functionalized dendrons by the reduction of the disulfide bonds at their cores [31]. A G6 cystamine-core PAMAM dendrimer was cleaved using tris(2-carboxyethyl)phosphine hydrochloride (TCEP) with ethylenediaminetetraacetic acid (EDTA). Sulfhydryl groups are stabilized by the presence of EDTA. SAM was formed using carboxy-EG_6_-undecanethiol on a sensor chip. The amine group of 3,3′-*N*-[*e*-maleimidocaproic acid] hydrazide (EMCH) was reacted with NHS esters on the carboxy-EG6-undecanethiol SAM after activation of the carboxyl groups by treatment with EDC/NHS. Then, the maleimide group of EMCH reacted specifically with the sulfhydryl groups of the dendron thiol. DNP–acetic acid was conjugated with the amine groups on the dendron surface after activation of carboxyl group of DNP–acetic acid with EDC/NHS.

TNT concentration was measured using an SPR immunosensor with the dendron-modified SAM surface. As a result, the binding capacity of the G6-dendron-immobilized surface to TNT–Ab was higher than those of the G3-dendron-immobilized surface and the surface containing no dendron. Forty ppb TNT–Ab was allowed to flow over the G6 dendron surface for 7 min. Nevertheless, a very low amount of TNT–Ab binding had occurred (approximately 90 RU) by the end of injection. An indirect competitive assay was conducted for TNT on the G6-dendron-immobilized surface. Although the maximum amount of binding was very low, the SD was also extremely low. The LOD of 15 ppt for TNT was finally realized.

#### 3.2.4. Surface Functionalization: Polymer

**Figure 5.**
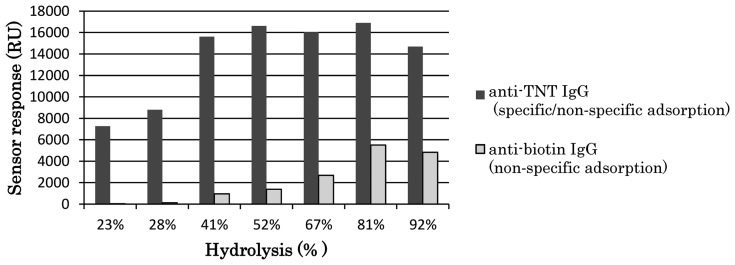
Hydrolysis dependence of specific/non-specific adsorption [40].

Poly-(vinylamine-*co*-*N*-vinylformamide) (poly-(VAm-*co*-NVF))-based SPR sensor chip supported by an alkanethiol SAM was developed and was used in the highly sensitive detection of TNT [40]. DNP–gly was bound to amino groups on the side chain of poly-vinylamine (poly-VAm) immobilized on a sensor chip. Poly-VAm can be obtained by hydrolyzing poly-vinylformamide [41]. High level of sensor response for binding of TNT–Ab to the sensor surface was obtained using the fabricated sensor chip. However, the response contained non-specific adsorption of TNT–Ab. Unreacted amino groups of the poly-VAm may be positively charged and cause non-specific adsorption due to electrostatic interaction. Thus, to control non-specific adsorption, the number of amino groups was reduced by making low-hydrolyzed poly-NVF, *i.e.*, poly(VAm-*co*-NVF) with various hydrolysis rates. Figure 5 shows the dependences of specific/non-specific adsorption of TNT–Ab and non-specific adsorption of anti-biotin antibody on each hydrolysis rate of poly-(VAm-*co*-NVF) used during the sensor surface fabrication. The LOD of 28 ppt for TNT was achieved using the sensor chip fabricated with 23% hydrolyzed poly-NVF (poly-[VAm-*co*-NVF]) by indirect competitive assay.

Surface-initiated atom transfer radical polymerization (SI-ATRP) has been employed for surface modification [42]. This polymerization method has the following characteristics. First, straight-chain polymers are directly linked to a surface because the polymer chains can grow from initiation points immobilized on the surface. Next, the polymerization reaction can be restarted because the growing points of the polymer chains are under a dormant condition even if the polymerization stopped. Namely, the polymerization can be restarted when required from the growing points of the polymer even after polymerization has stopped. It is easy to control the length of the polymer, and several types of polymers can catenate each other in series owing to these characteristics.

To achieve a surface able to suppress non-specific adsorption, SI-ATRP has been applied to fabricate zwitterionic polymer brushes. These brushes have previously been described to enable specific detection of proteins in blood plasma without purification [43]. As another application of SI-ATRP, two layers of polymer were fabricated in series from a surface to obtain both of low fouling property and high protein loading property by living polymerization [44]. Growth of polymer from antibody bound on a surface by ATRP was used for signal amplification of SPR measurement [45]. Thus, the SI-ATRP technique has been used for the fabrication of various types of surface.

**Figure 6.**
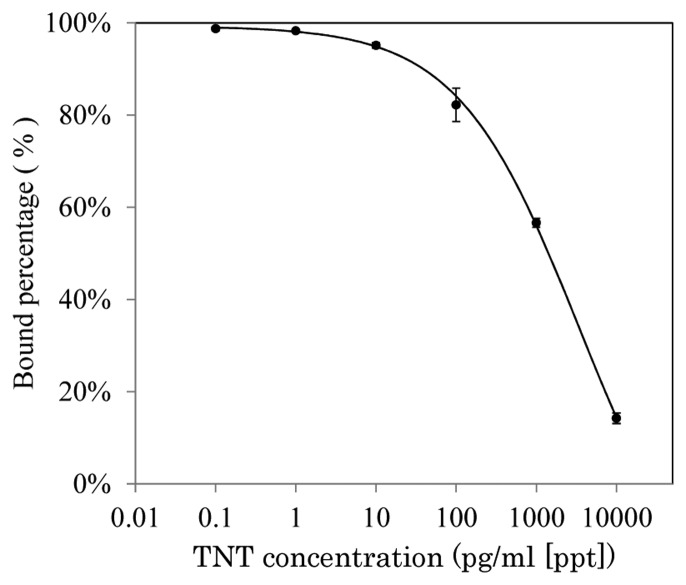
Response curve for TNT by inhibition assay using 100 ng/mL (100 ppb) TNT–Ab. The error bar refers to the SD [46].

**Scheme 3.**
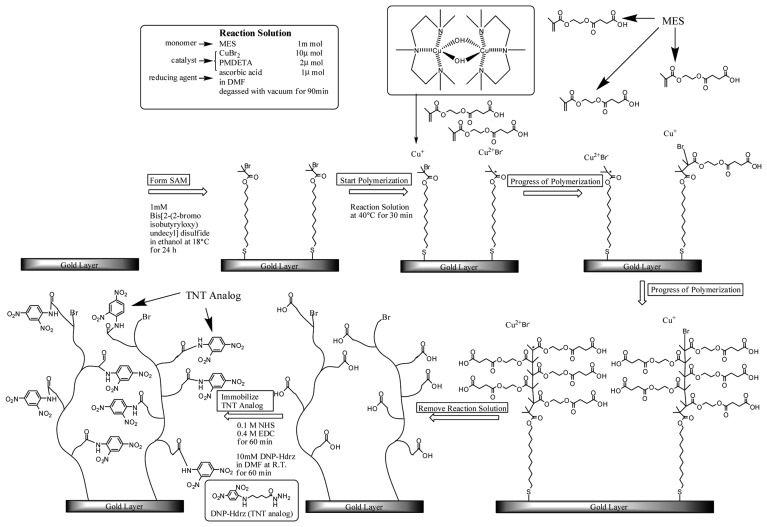
Fabrication process of polymer-based sensor surface by SI-ATRP [46].

We modified an SPR sensor surface with a polymer using SI-ATRP [46]. Scheme 3 shows the fabrication process of the sensor surface by SI-ATRP. To immobilize a TNT analog, 2,4-dinitrophenyl-*e*-aminocaproyl-NHNH_2_, on the polymer, mono-2-(methacryloyloxy)ethylsuccinate (MES), which has a carboxyl group, was used as a monomer. In this case, MES, which is negatively charged, may cause non-specific adsorption of TNT–Ab by an electrostatic interaction. Therefore, a mixed monomer of MES and diethylaminoethylmethacrylate (DEAEM), which has a tertiary amino group and is positively charged, was utilized to obtain electrically neutral property for avoiding the non-specific adsorption. The detection of TNT was carried out by indirect competitive assay using the polymer surface. The affinity between the surface and TNT–Ab was optimized by controlling the density of the initiator for ATRP by mixing two types of self-assembled monolayer reagents for improvement of the sensitivity to TNT. Figure 6 shows the response curve for TNT. A LOD of 5.7 pg/mL (ppt) for TNT was obtained using the optimized surface and TNT–Ab by indirect competitive assay. The SD on the surface at 0.1 ppt TNT was 0.9% and the LOD was calculated using three-fold SDs, equaling 2.7%.

### 3.3. Target: Research Department Explosive (RDX)

#### Surface Functionalization: OEG

**Figure 7.**
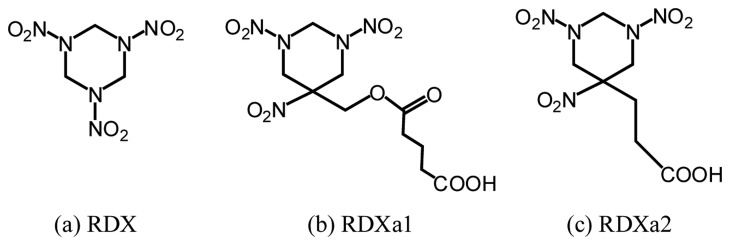
Structural formulae of (**a**) RDX, (**b**) RDXa1, and (**c**) RDXa2.

We developed a method of detecting 1,3,5-trinitroperhydro-1,3,5-triazine (research department explosive (RDX)) [16]. An anti-RDX monoclonal antibody was obtained from hybridoma cells that are prepared by the iliac lymph node method [47]. RDX solution and two RDX haptens, *i.e.*, (1,3,5-trinitrohexahydropyrimidin-5-yl) methylhemiglutarate (RDXa1; C_10_H_15_N_5_O_10_; molecular weight, 365.25) and 5-(2-carboxyethyl)-1,3,5-trinitrohexahydro pyrimidine (RDXa2; C_7_H_11_N_5_O_8_; molecular weight, 293.21), were prepared. Figure 7 shows the structural formula of RDX, RDXa1 and RDXa2 [48]. The immunogen was synthesized using RDXa1 and keyhole limpet hemocyanin (KLH). The dissociation constant of the antibody against RDX is estimated to be 9.6 nM from the results of indirect competitive enzyme-linked immunosorbent assay (ELISA). Table 1 shows the cross-reactivity of the antibody to RDX analogs and nitroaromatics.

We fabricated a sensor chip on which RDXa2 were immobilized through a SAM with OEG chains. High reliability and low false recognition rate are particularly required for sensors used for detecting explosives. Therefore, non-specific adsorption of the fabricated sensor chip is evaluated to confirm whether the sensor chip inhibits non-specific adsorption caused by electrostatic or hydrophobic interaction. It can be evaluated using bovine serum albumin (BSA) and lysozyme [49]. BSA was negatively charged, whereas lysozyme was positively charged in the HEPES-buffered saline (HBS, pH 7.4) used for measurement. It was confirmed that the sensor chip exhibited high ability to suppress non-specific adsorption. The response characteristics of the prepared antibody against RDX were examined by indirect competitive assay. To shorten total measurement time on the indirect competitive assay for RDX, requirement of incubation time was investigated. The LOD of 40 ppt was realized at an antibody concentration of 40 ppb and an incubation time of 0 min. There is a slight difference of detection limit between the condition under 0 min of incubation and under 15 min incubation. However, the characteristics of these curves were almost the same.

### 3.4. Target: Capsaicinoids

#### Surface Functionalization: OEG

Chemical warfare agents (CWAs) rapidly act on humans to kill or injure them even in small quantities. Large-scale terrorist attacks using CWAs (which are referred to as chemical terrorist attacks) may cause serious damage, and countermeasures based on scientific technologies are required. Lachrymators are one of the CWAs that could be used in terrorist attacks [50]. A lachrymator causes various indications such as pain, vomiting, a burning sensation, and breathing difficulty for people. One example of a lachrymator is capsaicin (oleoresin capsicum: OC) [51]. Some self-defense sprays contain below 10% OC, which consist of capsaicinoids and is extracted from *Capsicum* [52]. Capsaicin usually exists with dihydrocapsaicin in OC. Thus, detection methods of capsaicinoids are also needed for on-site detection.

We developed the detection method of capsaicinoids using an SPR immunosensor for on-site detection [53]. We paid attention to the fact that capsaicin is found with other capsaicinoids, such as dihydrocapsaicin, because these were extracted from *Capsicum*. A batch of capsaicin and dihydrocapsaicin, and their analogs, is appropriate for the analysis of collected samples in the field at initial investigation from the view point of sample concentration.

**Figure 8.**
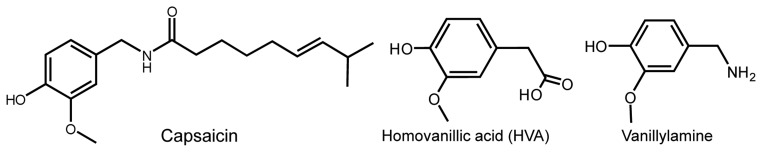
Structural formulae of capsaicin, homovanillic acid and vanillylamine.

To recognize a vanillyl group of capsaicinoids from a collected sample in a lump, a polyclonal antibody against homovanilic acid synthesized with *Concholepas concholepas hemocyanin* (CCH) was originally developed. We also prepared a sensor chip on which capsaicin analogs are bound via a SAM with OEG chains. An indirect competitive assay was performed to detect capsaicinoids using SPR sensor chips on which capsaicin analogs different from the immunogen were immobilized. Figure 8 shows the structures of capsaicinoids, homovanillic acid and vanillylamine (4-hydroxy-3-methoxybenzylamine) for the sensor chip on which vanillylamine was immobilized. The LOD of 150 ppb was accomplished by indirect competitive assay. It was found that the incubation time was not required. The detection was finished in five minutes and the regeneration time of the surface ready for the next sample was three minutes.

## 4. Assay Type: Displacement Assay

**Figure 9.**
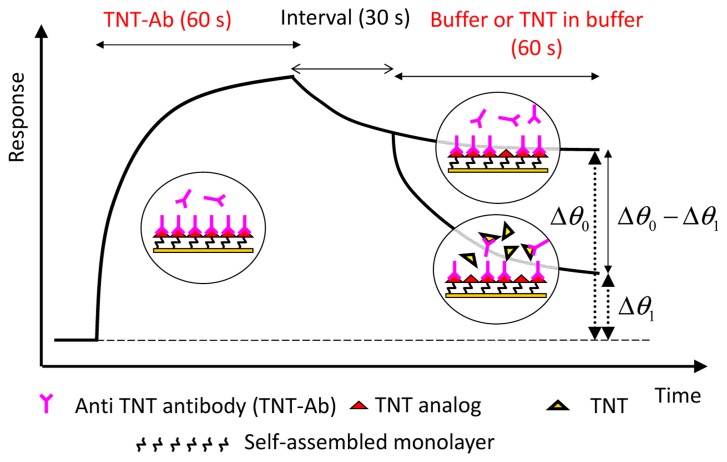
Principle of displacement assay on an SPR sensor.

A displacement immunoassay in continuous flow system does not require incubation [54]. An antibody is immobilized on a membrane or a column, and a fluorescent-labeled antigen is bound to the immobilized antibody before the antigen solution is injected. An antigen is introduced over the antibody, and the fluorescent-labeled antigen displaces the introduced antigen. Fluorescence intensity was detected at downstream of the flow line [55–57]. We applied displacement assay to the SPR sensing for rapid and sensitive detection of explosives. Figure 9 shows the principle of displacement assay for detection of TNT on an SPR sensor. The displacement ratio was defined by the equation (Δθ_0_ – Δθ_1_)/Δθ_0_ ·100 [3].

### 4.1. Target: TNT

Larsson *et al.* reported that TNT concentration can be measured by a displacement method based on SPR [58]. OEG–alkylthiols terminated with a hydroxyl group and TNT analogs were synthesized and the sensor surface was modified with mixed SAMs using the synthesized chemicals. An anti-TNT antibody was bound to the sensor surface and spontaneous dissociation was allowed until it became stable. Our sensor surface was similar; however, we took a slightly different approach than that described in Figure 9. Antibodies used in the measurement were divalent, and any antibody that interacted to one hapten had a very high possibility of interacting with a second hapten. However, when the concentration of the antibody flowing over the sensor surface is substantially high, it is thought that a univalent hapten–antibody complex will be formed because of the steric hindrance [59]. That is, the ratio of univalently interacting antibody will increase. After the injection of an antibody is finished without delay, univalently bound antibodies (with weaker interaction than divalently bound antibodies) spontaneously dissociate from the sensor surface. When the sensor response becomes stable, most of the remaining antibodies bound to the surface are divalently surface-bound antibodies. Therefore, we did not wait for spontaneous dissociation to become stable.

**Figure 10.**
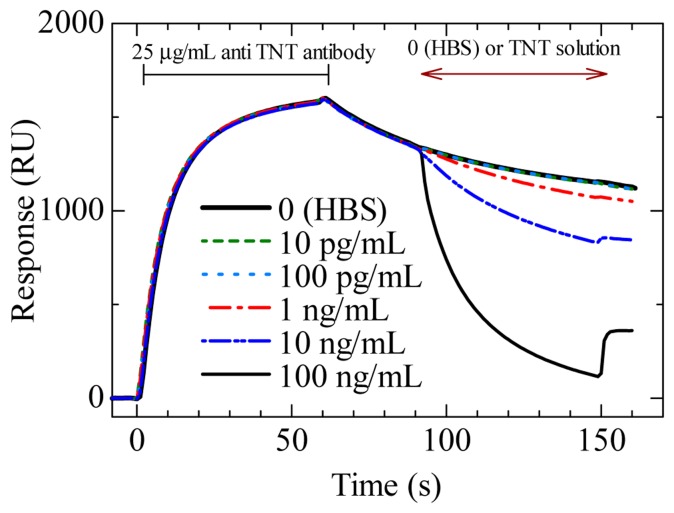
Sensorgrams of displacement immunosensor using 25 μg/mL TNT–Ab to detect varying concentrations of TNT on DNP–gly-modified sensor chip [60].

Figure 10 shows a sensorgram of displacement assay on an SAM surface containing DNP–gly immobilized with PEG_6_–COOH aromatic dialkanethiol [60]. The sensor surface was fabricated by a simplified version of the fabrication process of the SAM containing OEG as described in 3.2.2. After injecting a 25 μg/mL (25 ppm) TNT–Ab, there was a change in the sensor response induced by the binding of the TNT–Ab to the sensor surface, and the sensor response was almost saturated within 30 s. After the flow of the TNT–Ab, the sensor response gradually decreased owing to spontaneous dissociation. The response curves for TNT flows of 0–100 pg/mL completely overlapped. However, the response curve for a 1 ng/mL (ppb) TNT flow was clearly different from those for the 0–100 pg/mL (ppt) TNT flows, indicating that dissociation was promoted by TNT at concentrations of as low as 1 ng/mL. The response curves of 10 ng/mL (ppb) and 100 ng/mL (ppb) TNT generated about 26% and 66% displacements, respectively, compared with the response curve for injected HBS. In these cases, large reductions of responses were observed, and the duration of the flow of TNT solution was only 1 min. The slopes of the sensorgram 10 s after the start of TNT injections were different, especially between 0 and 100 ppb injection. Therefore, the slopes can be used with the aim of shortening the measurement time for detecting TNT.

**Figure 11.**
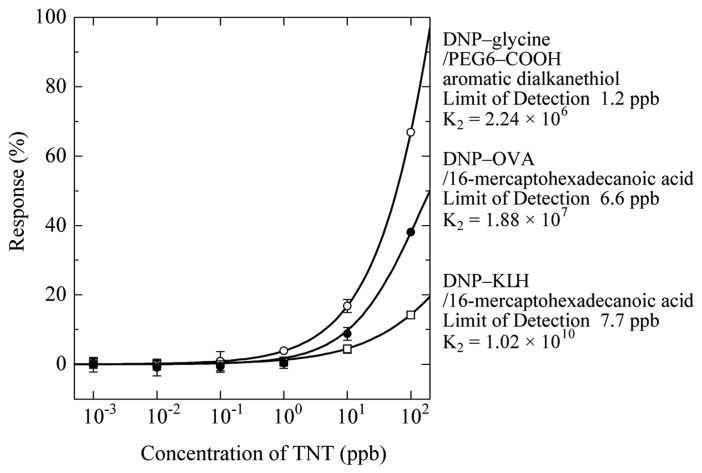
Response curves for TNT in the case of using three different antigen analogs [61].

Figure 11 shows a comparison of response curves for TNT on three types of sensor surfaces [61]. The DNP–gly sensor surface is the most sensitive to TNT among the three chips. DNP–ovalbumin (DNP–OVA) and DNP–KLH contain proteins and their structures are similar to the TNP–gly–KLH immunogen of TNT–Ab antibody. The displacement responses in these two sensor chips were lower than that for the DNP–gly sensor surface. LOD was about 1 ppb for 1 min flow of TNT solution.

### 4.2. Target: DNT

Detection of DNT by displacement assay was carried out using sensor chips modified with mixed SAMs containing OEG [62]. The SAM carboxyl terminal groups were reacted to amino terminal groups in mixture of ethylenediamine and ethanolamine with certain molar ratios (10:0, 7:3, 5:5). To immobilize DNP–gly to ethylenediamine, carboxyl groups of DNP–gly were activated using a mixture of EDC and NHS. Then the activated DNP–gly was reacted with the amino terminated SAM. Anti-DNT monoclonal antibody (DNT–Ab) was used to the assay [15].

**Figure 12.**
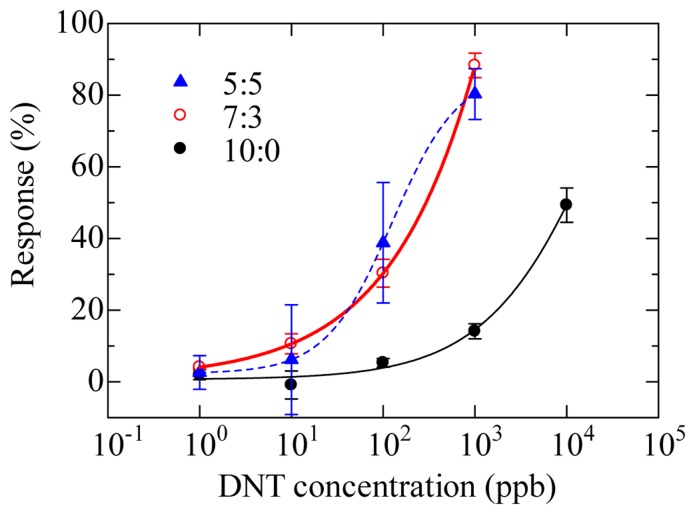
Response curves obtained by displacement assay of three ratios of DNP–gly-modified surfaces [62].

Figure 12 shows the response curves obtained by displacement assay of three ratios of DNP–gly-modified surfaces. The sensitivity on 7:3 ratio sensor chip is higher than 10:0 ratio sensor chip. When the density of the DNP–gly-modified surface is low, it is assumed that a monovalent antibody–hapten complex would form. Namely, the ratio of monovalently bound antibody would increase. In that case, a monovalently bound antibody is easy to dissociate from DNP–gly by associating with DNT. Finally, we obtained 8.8 ppb of LOD using 7:3 sensor chip.

Table 2 summarizes the LODs and the conditions for hazardous compounds by SPR sensing described in Sections 3 and 4.

## 5. Sampling Method

### 5.1. Preconcentrator

**Figure 13.**
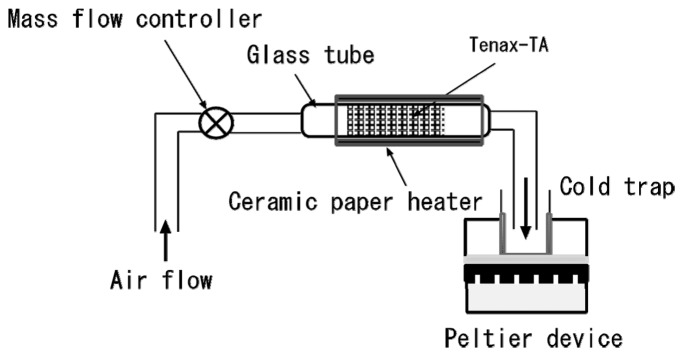
Schematic diagram of preconcentrator [63].

An explosive vapor diffuses from a bomb to the surrounding atmosphere. To use an SPR sensor system as an electronic dog nose at the secondary check of bomb detection after an initial check with a metal detector or another method, a vapor-sampling method was applied for the SPR sensor system. A preconcentrator, made up of a glass tube (internal diameter: 4 mm) filled with porous polymer beads as an adsorbent, was used in the sampling method for formation of a solution of nitroaromatic compounds [63]. The beads were Tenax-TA (60/80 mesh), which is commercially available and appropriate for concentrating volatile and semivolatile compounds. The amount of Tenax-TA used was 200 mg, and Tenax-TA was retained in the tube using silica wool. A schematic diagram of the preconcentrator is shown in Figure 13.

Dinitrobenzene (DNB), DNT, and TNT vapors were selected as target substances. One hundred ppm DNB solution, 100 ppm DNT solution, and 20 ppm TNT solution were used for generating their vapors. Five milliliters of each solution were evaporated by heating in a 1 L conical flask. To cool the vapors, the flask was left with lid on at room temperature for 30 min. Vapors of the aromatic nitro compounds were pumped from the flask to the preconcentrator as a flow controlled by a mass flow controller. The flow was stopped temporarily, and then heating started. A temperature of 300 °C was reached in 40 s using two ceramic paper heaters. The aromatic nitro compounds released from Tenax-TA were moved to a cold trap at a rate of 2 L/min for 30 s. The cold trap was a glass vial chilled by a Peltier device. The aromatic nitro compounds were dissolved in PBS for 20 s after pouring 100 μL of PBS into the vial. The concentrations of sample solutions were measured using an SPR immunosensor based on the indirect competitive assay. A commercially available anti-DNP–ovalbumin (OVA) monoclonal antibody and a DNP–OVA immobilized on CM5 sensor chip (GE healthcare) were used for the assay.

Table 3 shows the calculated concentrations of sampled explosives obtained using the indirect competitive assay on the SPR sensor. Percentages of binding were calculated as described in Section 3 of this review. The calculated concentrations were obtained using calibration curves for DNB, DNT, and TNT. The contact times of the target vapors with Tenax-TA at a lower flux were longer than those at a higher flux. The concentration of target substances adsorbed on Tenax-TA increased with decreasing flux, and the target substances were dissolved in a buffer solution. Therefore, target substances in buffer solutions that are sampled at a lower flux will have a higher concentration. Although we can collect explosives using the preconcentrator, considerable time is required to obtain a higher concentration. The total sampling time at the aspiration flux of 0.2 L/min was 450 s.

### 5.2. Wiping Method

**Figure 14.**
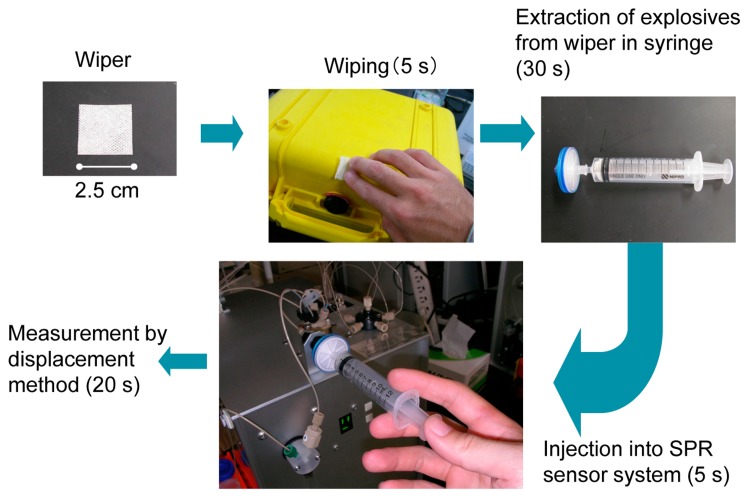
Procedure of wiping method [3].

The procedure of sample collection by the wiping method is shown in Figure 14. A suspected area is wiped using a wiper for about 5 s. The explosives are extracted from the wiper using a syringe with a filter [3]. The explosive in the syringe with the filter is extracted by an extraction buffer for about 30 s, and the solution is injected into the SPR sensor. The quantity of explosive is measured by displacement assay.

A portable SPR sensor (bottom center of Figure 14), which weighs about 10 kg, was developed and used for the measurement. The SPR sensor consists of an optical system in the Kretschmann configuration (770 nm LED light source, CMOS camera), a syringe pump, a flow cell, and an injector valve. The detection of TNT in 1 min using the prototype SPR sensor and the explosive sampling procedure was demonstrated at Central Customs Laboratory, Ministry of Finance, Japan [3,64]. The situation used in the demonstration was that of a TNT-contaminated suitcase. We chose the displacement assay and wiping methods to demonstrate rapid detection by the electronic dog nose as a first step.

**Figure 15.**
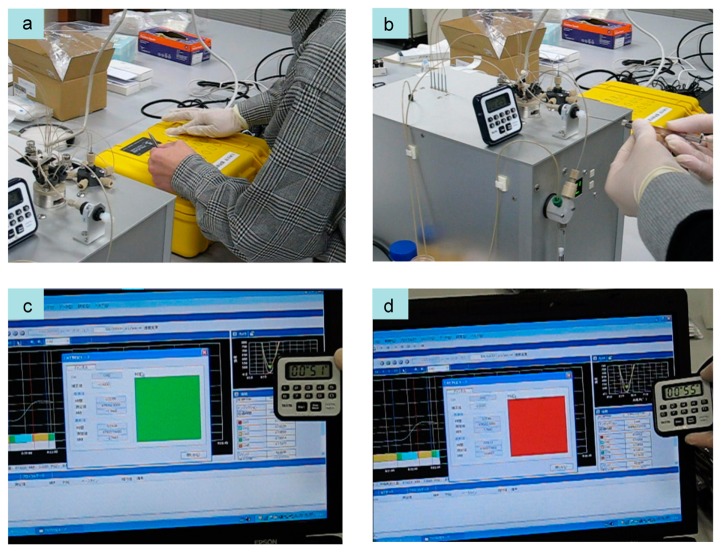
Demonstration process of TNT detection using the first prototype SPR sensor with displacement and wiping methods [64].

Figure 15 shows the process of TNT detection. TNT solution was added dropwise on a small plastic suitcase and was dried in advance. Wiping was carried out as shown in Figure 15a, and then TNT was extracted from the wiper using a buffer solution. The extracted solution was allowed to flow over the sensor surface on the SPR sensor, which had already been prepared by binding with the TNT–Ab, as shown in Figure 15b. The system software made a judgment based on the slope of the sensorgram during the decrease in antibody binding as described in Section 4.1. The threshold of the slope had been determined and set in the setting of the software in advance. Wiping and extraction required 20 s, and 15 s was needed for the solution to reach the flow cell of the prototype SPR sensor. Figure 15c shows the screen of the system software. The software displayed a green light at the center of the figure, with means that no explosive was detected. At this time, the timer displayed 51 s. Figure 15d shows a screen with a red light, meaning that an explosive was detected. At that moment, the timer displayed 55 s. Twenty seconds was required for the TNT solution to reach the flow cell from the injection port. Under the present circumstances, antibody binding and the regeneration process require an additional two minutes for the next measurement; however, the demonstration of TNT detection in 1 min using the prototype SPR sensor with the displacement method and wiping method was as successful as the first step of the electronic dog nose.

## 6. Clues for Improvement of Electronic Dog Nose

### 6.1. For Artificial Olfactory Mucosa

Functional polymers such as thermo-, pH-, light-, temperature-responsive polymers have been developed [65–69]. Antigen-responsive hydrogel, which swells to react a specific antigen in a buffer solution, is one of the polymers [70,71]. The hydrogel was linked by antigen–antibody interaction between antigen immobilized on the polymer chain and antibody immobilized on the polymer chain. If free antigen diffuses in the hydrogel, the antigen competes with the antigen immobilized on the polymer chain, and then the hydrogel swells. This mechanism is the same with the displacement assay as described above. A similar phenomenon was discovered for an SPR sensing for low-molecular weight substances using molecularly imprinted polymer with embedded Au-nanoparticles (AuNPs) [72].

### 6.2. For Artificial Olfactory Receptor

Benzaldehyde and furfural are typical fragrant compounds for peach-flavored beverages, and methyl anthranilate is a typical flavor of grape beverages. We can obtain antibodies against these fragrant compounds through the iliac lymph node method. Anti-fragrant monoclonal antibodies were developed [73], and an SPR measurement and open-sandwich enzyme-linked immunosorbent assay for benzaldehyde were carried out [74,75].

Triacetone triperoxide (TATP) is an easily obtainable explosive compared to TNT and RDX. Therefore, detection methods for TATP are required. Walter *et al.* developed a TATP analog to synthesize immunogen for generating antibodies in two mice. LOD for TATP in ELISA using sera obtained from immunized mice were 65 ng/mL and 870 ng/mL, respectively [76]. A thousand-fold lower LOD, 10 pg/mL, was achieved using sera obtained from immunized rabbits [77].

Normally, immunoglobulin G has two antigen-binding sites. Fab fragment can be prepared using papain and reducing agent. The increase in sensitivity to a target substance was observed when Fab fragments were used instead of whole IgG [78,79]. Liu *et al.* developed single chain variable fragment (scFv) of anti-TNT monoclonal antibody recombinantly. The ScFv was able to detect TNT 10-fold more sensitively than the monoclonal antibody [80].

Goldman *et al.* selected phage displayed peptides that bind to immobilized 2,4,6-trinitrobenzne in artificial seawater [81]. They found that a single good binder shares the consensus sequence His Arg at positions 2 and 3 of the amino terminal end of the fused 12-mer, and a Trp at position 9 in the 12-mer. They demonstrated an indirect competitive ELISA using the peptides and developed a fluorescent displacement immunosensor.

Walker *et al.* reported an integrated optical waveguide spectrometry with molecularly imprinted sol-gel sensing film for detection of gas-phase TNT [82]. A template was synthesized using 4-methyl-3,5-dinitrobenzyl alcohol and 3-isocyanatopropyltrimethoxysilane. The template was co-polymerized with 9:1 ratio of bis(trimethoxysilylethyl)benzene (BTEB) and 2-(trimethoxysilylethyl)pyridine (TMSEPyr). The template was removed and then a pore with a TNT shape having a primary amine group was created. The amine group can bind with deprotonated TNT. The LOD to gas-phase TNT was found to be 5 ppb using the sensing film on the waveguide.

Riskin *et al.* reported SPR sensing by a bis-aniline-cross-linked picric acid-imprinted AuNPs composite on an Au surface [83]. AuNPs modified with mixed monolayer of thioaniline and mercaptoethane sulfonate were cross-linked by electropolymerization with embedding picric acid. Picric acid was used as a template for imprinting of TNT molecular recognition site and was removed before association with TNT. The formation of the π–donor–acceptor complexes between TNT and the bis-aniline units of the composite triggered the changes of the dielectric properties of the composite. Enhancement of SPR sensitivity to TNT was enabled by following the changes in reflectance intensities of SPR deriving from the coupling of the localized plasmon of AuNPs with the surface plasomon wave associated with the Au surface. The LOD was 1.2 parts per quadrillion (ppq) for TNT using the proposed method. They also proposed the imprinted AuNPs composite for detection of RDX [84].

Gas-phase detection for large molecules using immobilized antibody on a solid interface were reported. Adachi *et al.* developed a method for the mass production of antibodies using the female ostrich (*Struthio camelus*) [85]. About 200 g of immunoglobulin yolk (Y) is obtained from only one ostrich in the course of a year. The ostrich has a life span of about 60 years and lays 100 eggs every year [86]. Kamiyama *et al.* confirmed that H5N1 avian influenza viruses were trapped in antibody (against H5N1 from ostrich eggs)-impregnated filters [87]. In addition, Iwanaga *et al.* found that an antibody immobilized on a solid interface can interact with airborne protein antigen with monitoring of fluorescence resonance energy transfer (FRET) [88]. These results indicated that the antibody can interact with the antigen under the gas-phase.

Fluorescent porous polymer films have also been developed for explosive detection. Fluorescence-quenching behavior of pentiptycene-derived phenyleneethynylene polymers can be used as TNT and explosive taggant dimethyldinitrobutane (DMNB) sensors. The transduction mechanism is a photoinduced charge transfer from the polymer donor to the nitroaromatic that binds via a tight π-complex to the polymer [89,90]. A similar concept of a fluorescent polymer for detection of explosives and taggants were also reported [91–95].

A graphite working electrode modified with a TiO_2_ thin film including single-walled carbon nanotubes (SWCNTs) on electrochemical measurement was proposed for highly sensitive and rapid detection of DMNB [96]. Incorporation of the SWCNTs into the inorganic matrix allowed the preparation of a highly stable film with an integrated electrochemical chip. Current change generated at the specific voltage attendant on the redox reaction of the nitro group in the DMNB was monitored. Under the optimized conditions, the linear range of the DMNB detection was 10 ppb to 10 ppm with a LOD of 12 ppb.

### 6.3. For Artificial Olfactory Nerve

Binding or adsorption of sample to the surface can be measured using SPR method without labeling, and hence the SPR method has been applied to the measurement of various biomolecular interactions. However, the SPR measurement is affected by non-specific adsorption and it is not easy to measure low-molecular weight substance with high sensitivity. On the other hand, the surface plasmon-field enhanced fluorescence spectroscopy (SPFS) method, which can selectively excite fluorescent molecules presenting at the interface using the surface field intensity enhancement produced by SPR, has been proposed [97–104]. We can obtain many advantages for sensitive detection of binding or adsorption of sample to the surface using SPFS, although antibodies have to be conjugated with fluorescent label.

Sensitivity enhancement methods of SPR biosensing for low-molecular weight substance using AuNPs conjugated with secondary antibodies has previously been reported [105,106]. Concentration of a primary antibody can be reduced using an AuNP-conjugated secondary antibody. Signal amplification method using nanobeads was also reported [107]. An immobilized antibody on an SPR sensor surface captured a target substance and a biotin-labeled antibody also interacted with the target substance captured by the immobilized antibody. Then, streptavidin-conjugated nanobeads interacted with bound biotin-labeled antibody. Furthermore, accumulation of biotin-labeled anti-streptavidin antibody and streptavidin-conjugated nanobeads were used for signal amplification.

Gold nanoparticle coated U-bend fiber optic probe was proposed for detection of TNT [108]. The AuNPs modified with L-cysteine and cysteamine exhibited a high selectivity towards TNT. L-cysteine on AuNPs formed Meisenheimer complex with TNT [109]. Cysteamine on AuNPs interacts with TNT by the donor–acceptor interaction. TNT is an electron acceptor and primary amine is a typical electron donor [110].

Mobile phones can be utilized for on-site sensing. Roche *et al.* proposed localized surface plasmon resonance (LSPR) biosensing method using a cell phone's camera [111]. A cuvette and a tricolor LED were mounted on the camera. Antibodies modified with AuNPs or gold nano rod were used in immunoassays. Absorbance change depending on the concentration of chemokine ligand 2 can be recorded with a LOD of 99 ng/mL. Preechaburana *et al.* have proposed an SPR optical system on cell phones [112]. The optical system was made with polydimethylsiloxane (PDMS) rubber. The cell phone's display and camera were utilized as a light source and detector for SPR monitoring, respectively. Mouse anti-human β_2_ microglobulin monoclonal antibody immobilized on Biacore sensor chip CM5 was set on the optical system for a demonstration. Interactions with 1.32 μg/mL and 0.132 μg/mL β_2_ microglobulin were observed on the system.

### 6.4. For Artificial Sniffing

King *et al.* reported the sampling performance of a commercially available batch-type wetted wall cyclone for bioaerosol sampling [113]. Aerosol enters a cyclone body tangentially through an inlet slot. A batch of liquid for collecting bioaerosol is injected at the start of the sampling cycle and the liquid spins inside the cyclone until the end of the cycle. The particles are hit on the inner wall, which is wetted by an air vortex acting on a liquid pool at the base of the cyclone.

Matsubara *et al.* reported a mist–cyclone system for odor sampling [114]. The mist–cyclone system has been developed for obtaining a high concentration solution of odor compounds by blowing an air mixture containing odor substances through the mist of water. The mist of water has an enormous contact surface generated by the ultrasonic transducer, and then separating the liquid from two-phase fluid by a cyclone unit. The amount of 0.06 ppm DNT in the air could be sampled. Similarly, a wetted wall cyclone using atomization air was also proposed [115].

## 7. Conclusions

Dogs trained for to search for explosives, landmines, drugs and human beings can recognize related chemical substances by olfaction. Odor-detection methods for detecting chemical substances that mimic organisms are expected to become more widespread. An electronic dog nose for explosive detection and its related technologies were introduced in this review.

Our research group mainly concentrated on developing sensor surfaces of the SPR sensor for developing the electronic dog nose. We aimed to use the electronic dog nose to detect explosives in the field. Therefore, we developed not only sensor surfaces, but also SPR instruments, sampling methods, antibodies, and the protocol for explosive detection.

We successfully demonstrated the detection of TNT in 1 min using the prototype SPR sensor as an electronic dog nose. The system is expected to be used for detection of drugs. Furthermore, the electronic dog nose can detect fragrant compounds like flavor of foods with high sensitivity and selectivity using antibodies against fragrant compounds. Odors of food and fragrances contribute to the quality of life. Moreover, we envisage that the electronic dog nose could be used for odor analysis of diseases such as cancer in the near future. Therefore, the concept underlying this sensor has the potential to contribute to an entirely new odor-sensing industry.

## Figures and Tables

**Table 1.** Cross-reactivity of rat anti-RDX monoclonal antibody [16].

Compounds	IC_50_ (M)	C.R (%)
RDX	1.56 × 10^−11^	100
RDXa1	1.49 × 10^−12^	1047
RDXa2	3.93 × 10^−11^	397
2,4,6-trinitrotoluene	9.94 × 10^−10^	1.57
1,3-dinitrotoluene	Not detected	–
2,4-dinitrotoluene	Not detected	–
2,6-dinitrotoluene	Not detected	–
2-amino-4,6-dinitrotoluene	Not detected	–
4-amino-2,6-dinitrotoluene	Not detected	–

**Table 2.** A summary of SPR immunoassays of explosives, capsaicinoids.

Assay Format	Target	Surface	Analog	Antibody	Antibody Concentration	LOD (ppt (pg/mL))	Ref. No.
Indirect competitive method	TNT	Dendrimer, SAM	DNP–KLH	Anti-TNT monoclonal (clone TNT A/1.1.1)	500 ppb	110	37
		OEG SAM	DNP–acetic acid		250 ppb	80	30
		Dendron, OEG	DNP–acetic acid		25 ppb	15	39
		Poly vinylamine-*co*-*N*-vinylformamide, SAM	DNP–glycine		100 ppb	28	40
		A polymer by SI-ATRP	2,4-dinitrophenyl-*e*-aminocaproyl-NHNH_2_		100 ppb	5.7	46
	DNT	OEG SAM	Dinitrobenzoic acid	Rabbit, Anti-DNP–KLH polyclonal (Kyushu Univ.)	20 ppm	20	13
	RDX	OEG SAM	5-(2-carboxyethyl)-1,3,5-trinitrohexahydro pyrimidine	Rat, Anti-RDX monoclonal (Kyushu Univ.)	40 ppb	40	16
	Capsaisinoids	OEG SAM	Vanillylamine	Rabbit, Anti-HVA-CCH polyclonal	25 ppm	150	53
Displacement method	DNT	OEG SAM	DNP-glycine	Rat, Anti-DNT monoclonal (Kyushu Univ.)	5 ppm	8800	62
	TNT	OEG SAM	DNP-glycine	Anti-TNT monoclonal (clone TNT A/1.1.1)	25 ppm	900	60
		SAM	DNP-OVA		100 ppm	6600	61
			DNP-KLH			7700	

**Table 3.** Percentages of antibody bound by indirect competitive assay and calculated concentrations of sample solution collected using preconcentrator [63].

Compound (Vapor Concentration)	Flux of Aspiration (L/min)	Percentage of Binding (%)	Estimated Concentration (ppb)
DNB (1180 ppb)	2.0	41.6	1500
	1.0	39.7	1800
	0.5	34.7	2800
	0.2	28.5	5700

DNT (140 ppb)	2.0	57.5	1200
	1.0	57.0	1300
	0.5	52.3	2200
	0.2	42.0	11,000

TNT (7.5 ppb)	2.0	52.9	210
	1.0	53.0	205
	0.5	50.6	250
	0.2	42.0	560
